# Treatment of Somatosensory Tinnitus: A Randomized Controlled Trial Studying the Effect of Orofacial Treatment as Part of a Multidisciplinary Program

**DOI:** 10.3390/jcm9030705

**Published:** 2020-03-05

**Authors:** Annemarie van der Wal, Sarah Michiels, Paul Van de Heyning, Marc Braem, Corine M. Visscher, Vedat Topsakal, Annick Gilles, Laure Jacquemin, Vincent Van Rompaey, Willem De Hertogh

**Affiliations:** 1 Department of Rehabilitation Sciences and Physiotherapy, Faculty of Medicine and Health Sciences, University of Antwerp, 2610 Edegem, Belgium; sarah.michiels@uantwerpen.be (S.M.); willem.dehertogh@uantwerpen.be (W.D.H.); 2 Department of Otorhinolaryngology, Antwerp University Hospital, Faculty of Medicine and Health Sciences, University of Antwerp, 2650 Edegem, Belgium; paul.vandeheyning@uza.be (P.V.d.H.); vedat.topsakal@uza.be (V.T.); annick.gilles@uza.be (A.G.); laure.jacquemin@uza.be (L.J.); vincent.vanrompaey@uza.be (V.V.R.); 3 Department of Translational Neurosciences, Faculty of Medicine and Health Sciences, University of Antwerp, 2610 Edegem, Belgium; 4 Multidisciplinary Motor Centre Antwerp, University of Antwerp, 2610 Edegem, Belgium; 5 Lab Dental Materials, University of Antwerp, 2610 Edegem, Belgium; marc.braem@uza.be; 6 Special Care Dentistry, University Hospital Antwerp, 2610 Edegem, Belgium; 7 Faculty of Medicine and Health Sciences, University of Antwerp, 2610 Antwerp, Belgium; 8 Department of Oral Health Sciences, Academic Centre for Dentistry Amsterdam, University of Amsterdam and VU University Amsterdam, Research Institute MOVE Amsterdam, 1012WX Amsterdam, The Netherlands; c.visscher@acta.nl

**Keywords:** tinnitus, temporomandibular disorders, occlusal splints

## Abstract

Background: Tinnitus, or ringing in the ears, is a perception of sound in the absence of overt acoustic stimulation. In some cases, tinnitus can be influenced by temporomandibular somatosensory input, then called temporomandibular somatosensory tinnitus (TST). It is, however, not entirely known if orofacial treatment can decrease tinnitus severity. The purpose of this study was to evaluate the effect of orofacial treatment on tinnitus complaints in patients with TST. Methods: Adult patients with TST were included, and all patients received information and advice about tinnitus and conservative orofacial treatment consisting of physical therapy, and, in case of grinding, occlusal splints were applied. Included patients were randomly assigned to an early start group and a delayed start group according to our delayed treatment design. Results: In total, 40 patients were included in each group. The treatment effect on tinnitus severity was investigated using the tinnitus questionnaire (TQ) and Tinnitus Functional Index (TFI). Regarding the TQ score, no clinically relevant reductions were observed, and no significant differences in the decrease were observed between the early start group and delayed start group. Contrarily, a significantly higher percentage of patients showed a decrease in the TQ degree in the early start group compared to the delayed start group (30.0% versus 2.8%, *p* = 0.006). The TFI score did show a significantly greater and clinically relevant reduction in the early start group compared to the delayed start group (*p* = 0.042). Conclusion: A multidisciplinary non-invasive orofacial treatment was able to reduce tinnitus severity in patients with temporomandibular related somatosensory tinnitus.

## 1. Introduction

Tinnitus, or ringing in the ears, is a perception of sound in the absence of overt acoustic stimulation. It occurs in 10% to 15% [1] of the adult population and is generally described as hissing, sizzling, or ringing, and can be constant or intermittent, located in one or both ears, or centrally located inside the head [1].

The international tinnitus guidelines distinguish between two forms of tinnitus, namely objective and subjective tinnitus [2]. Tinnitus is “objective” when the tinnitus sound originates from an internal source within the patient’s body, such as turbulences in the blood flow [1]. In the case of subjective tinnitus, on the other hand, no source (externally or internally) can be found for the perceived tinnitus [1]. Subjective tinnitus can have many different etiologies and often has a multifactorial origin with several influencing factors. Common influencing factors are hearing loss, noise trauma, or psychological factors, such as stress, depression, and anxiety disorders. Additionally, altered somatosensory input from the cervical spine or temporomandibular area can also influence the tinnitus perception [1,3,4]. When this somatosensory influence is one of the major influencing factors, a patient’s tinnitus is called somatic or somatosensory tinnitus (ST). This type of tinnitus is present in 12% to 43% of tinnitus patients, depending on the setting (primary care, tertiary care, open population) and diagnostic criteria for ST [5,6,7,8].

Physiologically, ST is explained by the presence of connecting fibers between the somatosensory system of the cervical spine and temporomandibular area and the dorsal cochlear nucleus (DCN). Through these fibers, afferent somatosensory information from the cervical spine or temporomandibular area can alter the spontaneous firing rates and synchrony of firing among neurons in the DCN, inferior colliculus, and auditory cortex. In this way, the somatosensory system is able to alter the pitch or loudness of an existing tinnitus or, in rare cases, it can also cause the tinnitus [9].

One reason for altered somatosensory input is the presence of temporomandibular disorders (TMDs) or oral parafunctions [6]. TMD comprises various conditions where pain and dysfunction of the masticatory muscles or the temporomandibular joint (TMJ) are involved [10]. Oral parafunctions (e.g., bruxism, excessive gum chewing, lip or fingernail biting) are often related to TMD [11,12]. The fact that prevalence studies show that tinnitus occurs in 30% to 64% [13,14] of patients with TMD, suggests that TMD and tinnitus are intertwined. Additionally, TMD and oral parafunctions are related to 5 out of 16 criteria that are part of the internationally agreed set of diagnostic criteria for ST [6].

Previous studies [15,16,17] showed that TMD treatment can positively affect tinnitus loudness and severity, but a high risk of bias is present in these studies. The main methodologic limitations in these studies were related to a lack of statistical analysis between groups, incomplete presentation of the data, and selective reporting [18]. Furthermore, a lack of blinding of subjects, therapists, and investigators caused a high risk of bias [19]. On the other hand, previous studies often used a primary TMD population to investigate the effect of TMD treatment on concomitant tinnitus complaints. Therefore, more high-quality research, with limited risk of bias, in a primary tinnitus population is necessary. Thus, the aim of this study was to investigate the effect of an evidence-based conservative orofacial treatment on subjective tinnitus complaints in patients with TMD-related ST while limiting the risk of bias.

## 2. Methods

### 2.1. Patients

Patients were recruited by the multidisciplinary team of the tertiary tinnitus clinic of the Antwerp University Hospital in Belgium. Patients were thoroughly assessed by the multidisciplinary team (with otorhinolaryngologists, audiologists, physical therapists, and dentists) to identify the influencing factors of their tinnitus and to exclude any objective causes [20]. Patients were included in the study when suffering from a combination of moderate to severe chronic subjective tinnitus, defined as a Tinnitus Functional Index (TFI) score between 25 and 90 [21], that had been stable for at least three months and TMD (diagnosed according to the diagnostic criteria for TMD [10]) and/or oral parafunctions. Patients suffering from otological or neurological causes of tinnitus, such as Menière’s disease, progressive middle ear pathology, intracranial pathology, severe depression or anxiety disorders (diagnosed by a psychiatrist), traumatic cervical spine or temporomandibular injury in the past 6 months, tumors, or previous surgery in the orofacial area, were not considered for inclusion. Patients were also excluded if they had received TMD treatment in the past three months.

### 2.2. Intervention

#### 2.2.1. Information and Advice

In our clinic, all patients receive information and advice concerning their tinnitus prior to any other treatment suggestion. This information and advice was provided by experienced clinicians in tinnitus treatment. 

#### 2.2.2. Orofacial Treatment

Orofacial treatment was provided by a team of dentists and physical therapists who were specifically trained to apply the required treatment prior to the start of the study. The intervention consisted of orofacial physical therapy, comprising counselling regarding mouth habit reversal, bruxism, sleep hygiene, lifestyle advice and biofeedback; massage of the masticatory muscles; stretching exercises; and relaxation therapy. In the case of grinding, the orofacial physical therapy was complemented with an occlusal splint by the dentist. In the case the patient also suffered from cervical spine problems, which is highly prevalent in patients with TMD [22,23,24], additional cervical spine treatment (mobilizations and exercises) was added to the treatment. This type of multidisciplinary orofacial treatment is currently the evidence-based treatment for the conservative management of TMD [25,26]. 

The treatment protocol provided a maximum of 18 orofacial physical therapy sessions during a 9-week treatment program as described in the published protocol [27].

The therapists adapted the used techniques and exercises to the patient’s current dysfunction, as this is the current best practice in orofacial treatment. No additional tinnitus treatments were allowed during the participation in the study.

### 2.3. Study Design

The study was designed as a randomized controlled trial with a delayed treatment design to evaluate the effectiveness of a conservative orofacial treatment on tinnitus annoyance in patients suffering from temporomandibular-related somatosensory tinnitus. The delayed treatment design allowed us to obtain data for a control group by creating a waiting list, since the use of a control group that receives no treatment at all was not considered ethical in a tertiary center population.

At baseline, patients were randomly assigned to receive immediate treatment (early start group) or to be placed on the waiting list (delayed start group). In phase 1 (weeks 0–9), the early start group received the orofacial treatment for 9 weeks. The delayed start group entered a wait-and-see period. In phase 2 (weeks 9–18), the patients in the delayed start group started their 9-week orofacial treatment period while the early start group entered a 9-week follow-up period. In phase 3 (weeks 18–27), the delayed start group entered their 9-week follow-up period. The early start group ended their participation in the study at the end of week 18.

### 2.4. Outcome Measures

#### 2.4.1. Primary Outcome Measure

The primary outcome measure for this study was the tinnitus questionnaire (TQ). The TQ is a validated and commonly used instrument for assessment of tinnitus annoyance. The TQ incorporates scales evaluating emotional and cognitive distress, intrusiveness, auditory perceptual differences, sleep disturbances, and associated somatic complaints. The TQ consists of 52 questions that are answered on a 3-point scale, ranging from ‘true’ (scoring 0), ‘partly true’ (scoring 1), to ‘not true’ (scoring 2). The total score on the TQ ranges from 0 to 84. Additionally, the total score can be used to create four groups based on the degree of tinnitus-related distress: Degree 1 (slight) up to 30 points, degree 2 (mediocre) between 31 and 46 points, degree 3 (severe) between 47 and 59 points, and degree 4 (extremely severe) between 60 and 84 points. The TQ showed a good correlation with the Tinnitus Handicap Inventory, Tinnitus Impairment Questionnaire, and Tinnitus Functional Index (0.79–0.90) [28,29]. A decrease of 8.72 points is considered clinically relevant [28]. 

#### 2.4.2. The Secondary Outcome Measures 

The Tinnitus Functional Index (TFI) was used to measure change in tinnitus severity after treatment and follow-up. This questionnaire consists of 25 questions divided into eight subscales: Intrusiveness, sense of control, cognitive, sleep, auditory, relaxation, quality of life, and emotion. For each question, patients respond on a Likert scale of 0–10, allowing the detection of small changes over time. The global score ranges from 0 to 100, with higher scores denoting higher levels of handicap. The test-retest reliability of the TFI is good (r = 0.78). The convergent validity with the Tinnitus Handicap Inventory (r = 0.86) and Visual Analogue Scale (VAS) (r = 0.75) is good. A reduction of 13 points is considered clinically relevant [21].

**Figure 1:**
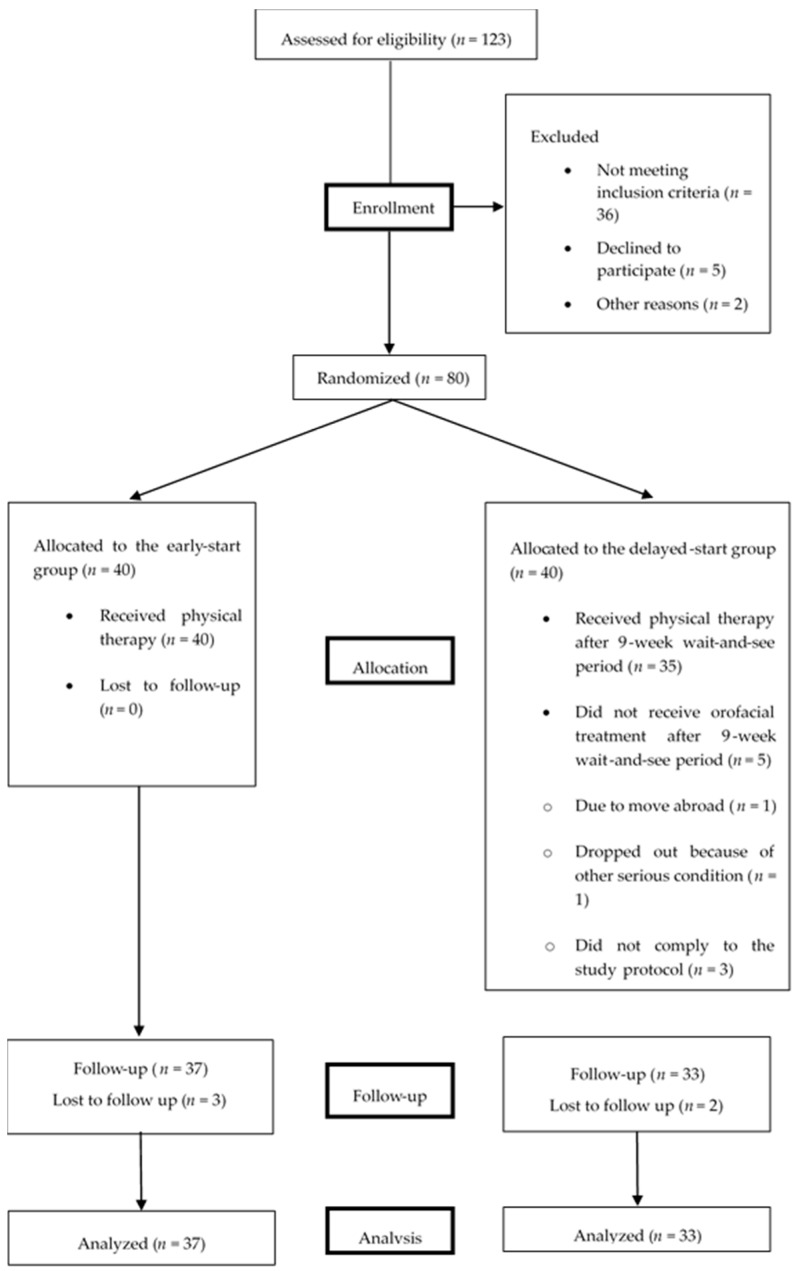
Flow chart of participation of the patients through the study.

All outcome measures were documented at baseline, after 9 weeks in the delayed start group, immediately after the last treatment session (post-treatment) and after 9 weeks of follow-up (Figure 1). 

More information about the used baseline measures is provided in the published protocol [7]. 

### 2.5. Sample Size and Power

The sample size was calculated using Medcalc (Medcalc Software bvba, version 6, Ostend, Belgium). Sample size calculation was performed for the clinically relevant change of 8.72 points in the TQ score [26]. The sample size was calculated for the study to have 80% power to reject the null hypothesis. The type I error probability, associated with this test, is 0.05. To achieve 80% power, 37 patients were required in each study arm. 

### 2.6. Randomization and Blinding

After baseline measurements, patients were randomized into the early start group or delayed start group based on block-randomization 1:1, with variable block lengths. A concealed randomization list was generated using Microsoft Excel® software by an independent researcher. Treating therapists were blinded at all times to whether a patient was included in the early start or delayed start group.

### 2.7. Ethical Approval and Consent

Ethical approval was obtained from the ethics committee of the Antwerp University Hospital (reference number: B300201730825, date: 9 January 2017). Written informed consent was obtained for all patients prior to the start of the study. The study was registered at ClinicalTrials.gov (NCT03209297).

### 2.8. Statistics

An intention-to-treat analysis was performed on the study cohort. First, the normality of the data was investigated using a Kolmogorov–Smirnov test. The baseline comparability (*p* > 0.05) of both groups was analyzed using descriptive statistics, Mann–Whitney U-tests for non-normally distributed data, and independent samples *t*-tests for normally distributed data. The chi square test were used to determine differences between dichotomous variables.

To answer our primary research question, differences in changes on TQ and TFI from baseline to week 9 of the study between the early start and delayed start groups were calculated to investigate the effect of orofacial treatment. The data in week 18 of the study were analyzed to investigate if a similar decrease in changes on the TQ and TFI score was present in the delayed start group after receiving orofacial treatment.

Before starting the actual analysis of the treatment effect, new variables were calculated, being the change in the TQ score, change in the TQ degree, and change in the TFI score from baseline to week 9 and from baseline to week 18. These parameters enabled us to compare the evolution of the TQ and TFI in both groups. Since the TQ change was not normally distributed, differences between both groups were compared using the Mann–Whitney U-test. Differences in the TFI change were calculated using the independent sample t-test. Additionally, for each patient, the TQ degree at baseline and at 9 weeks was determined according to the guidelines of Zeman et al. [28]. The change in the TQ degree was calculated as the difference between the TQ degree at baseline and the TQ degree in week 9 of the study. Differences between both groups in change in the TQ degree were compared using the Mann–Whitney U-test. 

In a third stage of the analysis, the TQ and TFI change scores were dichotomized, based on the clinically relevant change of 8.72 points for the TQ and 13 points for the TFI [21,28]. Differences in the number of patients who perceived a clinically relevant change in both groups were calculated using chi square tests.

Additionally, within-group differences from baseline to post-treatment and follow-up were analyzed using paired sample tests. Hereby, we expected that the TQ score and TFI score in the early start group would decrease from baseline to week 9 while the delayed start group would stay stable or have a slight decrease (because only the early start group received orofacial treatment). At the end of phase 2, these differences between the two groups would become smaller, because the delayed start group also received orofacial treatment in that period and the early start group ended the treatment. 

## 3. Results

### 3.1. Patients

In total, 80 patients were included in the study over a period of 2 years: 40 patients were randomly assigned to the early start group and 40 patients to the delayed start group. In the early start group, all 40 allocated patients received orofacial treatment. Three of them were lost to follow-up after the 9-week follow-up period. In the delayed start group, 35 patients received orofacial therapy after the 9-week wait-and-see period. Reasons for drop-out are specified in Figure 1. Two of the treated patients were lost to follow-up after the 9-week follow-up period. An overview of the enrolment is shown in Figure 1. In addition to the physical therapy, 52% of the patients (*n* = 39) received an occlusal splint. These patients were equally distributed over both groups. 

The baseline characteristics of the patients in the two groups are presented in Table 1. Both groups were similar in terms of clinical and demographic characteristics. More specifically, no significant differences in baseline characteristics (Tinnitus Functional Index (TFI) score, age, and gender) were found between both groups (Table 1).

### 3.2. TQ Responses to Treatment (Primary Outcome)

**Figure 2:**
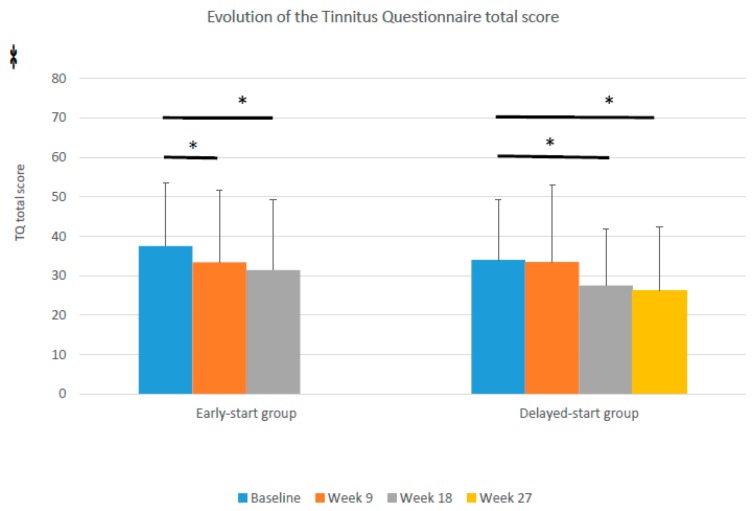
Evolution of the tinnitus questionnaire (TQ) scores in the early start and delayed start groups (error bars show the standard deviations, * *p* < 0.05).

The TQ score in week 9 of the study showed a decrease of 4.1 points in the early start group, compared to 0.2 in the delayed start group. This difference in the decrease between both groups, however, was not statistically significant (Mann–Whitney U (Z)= 561.5 (−1.650), *p* = 0.099) nor clinically relevant (decrease < 8.72 points). A comparable decrease (6.0 points) was found after treatment in the delayed start group (week 18). Both groups showed an additional decrease in the TQ score after follow-up: 2.0 points in the early start and 1.2 points in the delayed start group. Figure 2 shows the difference in the evolution between the early start and delayed start groups. 

Looking at the within-group analysis, the early start group showed a significant decrease in the TQ score from baseline to 9 weeks (*t* (df) = 2.206 (39) *p* = 0.033), whereas the delayed start group remained stable (*t* (df) = 0.158 (35) *p* = 0.875). In week 18, after completion of the orofacial treatment in the delayed start group, a significant decrease in the TQ score was found from baseline to 18 weeks in the delayed start group (*t* (df) = 2.717 (30) *p* = 0.011). The decrease in both groups after follow-up did not reach the clinically relevant reduction of 8.72 points. 

The TQ degree in week 9 of the study did differ significantly (Mann–Whitney U (Z) = 506.5 (−2.740) *p* = 0.006) between both groups. In the early start group 30.0% of the patients showed a decrease of at least one degree on TQ, compared to 2.8% in the delayed start group. 

### 3.3. TFI Responses to Treatment (Secondary Outcome)

The TFI scores in week 9 showed a decrease of 13.8 points in the early start group and 5.0 points in the delayed start group. This difference in the decrease was significantly different (*t* (df) = −2.073 (75) *p* = 0.042), indicating a significant effect of our treatment on tinnitus severity. This effect was bolstered by equivalent decreases in the TFI score after completion of the treatment in the delayed start group (12.2-point reduction). 

**Figure 3:**
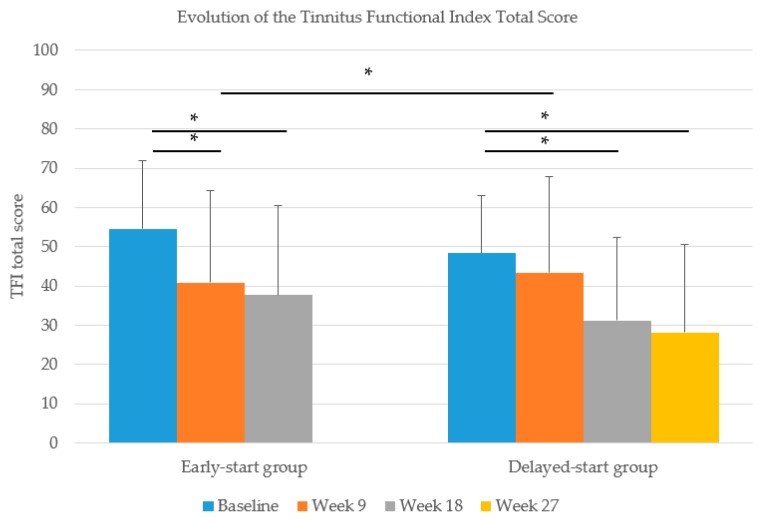
Evolution of the Tinnitus Functional Index (TFI) scores in the early start and delayed start groups (error bars show the standard deviations, * *p* < 0.05).

Both groups showed an additional decrease in the TFI score after follow-up: 3.1 points in the early start and 3.0 points in the delayed start group. A clinically relevant reduction of 13 points was found after treatment in the early start group and after follow-up in both groups. Figure 3 shows the difference in the evolution between the early start and delayed start groups. 

Looking at the within-group analysis, the early start group showed a significant decrease in the TFI score from baseline to 9 weeks (*t* (df) = 4.254 (38) *p* = 0.000) while no such decrease was found in the delayed start group (*t* (df) = 1.825 (37) *p* = 0.076). Although, after completion of the orofacial treatment in week 18, a significant decrease in the TFI score compared to baseline was found in the delayed start group (*t* (df) = 3.693 (29) *p* = 0.001).

### 3.4. Clinically Relevant Improvement

For the entire group, 34% of the patients showed a clinically relevant improvement of the TQ score (cutoff score ≥ 8.72 points [28]) immediately after treatment. Looking at the TFI score, 41% of the patients had a clinically relevant improvement (cutoff score ≥ 13 points [21]). After the follow-up period 46% (TQ) and 61% (TFI) reached the level of clinically relevant improvement compared to baseline, respectively.

## 4. Discussion

To investigate the effect of a non-invasive orofacial treatment on tinnitus annoyance, the current study was designed using a delayed treatment design. A significant difference in the decrease in the TQ degree was found between the early start and delayed start group, as well as a significant decrease in the TFI score, indicating a positive effect of orofacial treatment on tinnitus severity. These results confirm the findings of previous RCTs by Bösel et al [17], Erlandsson et al. [15], and Tullberg et al. [16] that were assembled in a recent systematic review [19]. 

After receiving orofacial therapy, a clinically relevant change in the TQ score was found in 34% of patients immediately after treatment and in 46% after follow-up. On TFI, larger percentages of clinically relevant change were found: 41% and 61% of patients immediately after treatment and after follow-up, respectively. This difference might be explained by differences in the scoring and construct of both questionnaires. The TQ uses a 3-point scale to rate each question while the TFI uses an 11-point Likert scale. This makes the TFI potentially more sensitive than the TQ to smaller differences in tinnitus severity. This difference in sensitivity to change was also pointed out by Jacquemin et al. [29]. On the other hand, the TQ is designed to measure tinnitus annoyance, whereas the TFI is used to measure tinnitus severity. Another reason for the differences in the results between the TFI and TQ scores might be our inclusion criteria. Patients were included when they had a TFI score between 25 and 90 points, but some patients already had a rather low TQ score at baseline. Hence, measuring a TQ change of more than 8.72 points might have been more difficult. 

Regarding the potential risk of bias, we believe that our study has a lower risk than those included in the abovementioned review, with only ‘blinding of subjects’ and ‘blinding of therapists’ that might have introduced a slight bias, which cannot be eliminated in this type of study. The current design and study results reinforce the usefulness of orofacial treatment in patients with temporomandibular-related somatosensory tinnitus.

Patients further improved after the TMD treatment was stopped, as was noted in an additional decrease in the TQ and TFI scores between the post-treatment and follow-up measurements, which is in line with previous studies on tinnitus treatment [30,31]. This might be due to the fact that patients continue their exercises and habit reversal after the last treatment session. The after-effect might also be due to the fact that patients are less focused on their tinnitus during the follow-up period than during the treatment period, as there is no more therapist contact [31]. 

The current study used a delayed treatment design, because the use of a control group that received no treatment was found to be unethical. Moreover, no comparable temporomandibular treatment that could serve as a sham intervention is available. The delayed treatment design, however, has some downsides. The most important one is the fact that we could only compare our groups in week 9 of the study while the largest effect of the treatment was found after follow-up. This might have caused an underestimation of the effect of our treatment compared to the wait-and-see period. A second disadvantage of the delayed treatment design is the lack of a comparison treatment to rule out placebo effects. Future research should compare the orofacial treatment to other treatments using two-armed randomized controlled trials.

We recruited patients from a tertiary tinnitus clinic. Patients in these settings are known to be more therapy resistant than patients in primary or secondary care. This might lead to an underestimation of the overall treatment effect. It would therefore be interesting to investigate the effect of our treatment in a primary or secondary care population in the future.

Additionally, we compared the effect of a single session of “information and advice” with a maximum of 18 sessions of orofacial treatment. Since the duration of patient–therapist contact can have an influence on the effect of the treatment, the chosen design might also have introduced an overestimation of our treatment effect. To rule out this potential over- or underestimation, future studies should focus on comparing the effect of orofacial treatment to other types treatments.

The randomization procedure using a 1:1 block randomization with variable block lengths was used to reduce bias and achieve balance in the allocation of patients between the early start and delayed start group. The lack of stratification on the baseline severity of tinnitus (TFI scores) might have influenced our results though and is a limitation of our study. In future studies, different randomization procedures should be used, stratifying for baseline TFI scores.

## 5. Conclusions

A multidisciplinary non-invasive orofacial treatment showed positive effects on tinnitus severity, compared to a single session of information and advice. This effect can be expected in patients with temporomandibular-related somatosensory tinnitus.

## Figures and Tables

**Table 1:** Baseline characteristics of the early start group and the delayed start group.

Characteristic	Early-Start Group	Delayed-Start Group	Total	*p* Value	Statistic Test	Value	Df
Number of subjects	40	40	80				
Gender male/female	18/22 (45%/55%)	24/16 (60%/40%)	42/38 (53%/47%)	0.168	Chi-square	1.805	1
Age in years (SD)	46 (13)	45 (15)	45 (14)	0.769	*t*-test	0.294	78
TQ (SD)	37 (16)	34 (15)	36 (16)	0.345	*t*-test	0.951	75
TFI (SD)	55 (17)	48(15)	52 (16)	0.086	*t*-test	1.741	78
TQ degree							
% degree 1	30%	49%	39%				
% degree 2	40%	30%	35%	0.376	Chi-square	3.105	3
% degree 3	15%	13%	14%				
% degree 4	15%	8%	12%				
VAS mean loudness tinnitus right ear (SD)	50 (30)	49 (25)	49 (27)	0.835	*t*-test	0.209	78
VAS mean loudness tinnitus left ear (SD)	46 (31)	45 (26)	45 (28)	0.858	*t*-test	0.180	78
TMD pain screener (percentage < 3/percentage ≥ 3)	35%/65%	35%/65%	40%/60%	0.272	Chi-square	7.561	6
HQ (SD)	17 (8)	19 (9)	18 (8)	0.958	*t*-test	−0.053	76
HADS anxiety (SD)	9 (4)	8 (4)	9 (4)	0.076	*t*-test	1.695	77
HADS depression (SD)	7 (5)	5 (4)	6 (5)	0.257	*t*-test	1.429	75
Fletcher index left (SD)	13 (17)	11 (11)	13 (17)	0.725	*t*-test	0.135	78
Fletcher index right (SD)	11 (10)	13 (14)	11 (12)	0.359	*t*-test	−1.254	76

SD: standard deviation. TQ: Tinnitus Questionnaire. TFI: Tinnitus Functional Index. VAS: Visual Analogue Scale. TMD: Temporomandibular Disorder. HADS: Hospital Anxiety and Depression Scale. HQ: Hyperacusis questionnaire.
